# Surgery time for stenosed Crohn's disease: Case report

**DOI:** 10.1016/j.ijscr.2025.110903

**Published:** 2025-01-20

**Authors:** Mohammad N. Nofal, Ali J. Yousef, Saad H. Samarah, Baidaa M. Al-Qudah

**Affiliations:** a General Surgery and Anesthesia Department, Faculty of Medicine, Mutah University, Karak 61710, Jordan; b General Surgeon, Arab Medical Center, Jordan; c Ministry of Heath-Jordan, Jordan

**Keywords:** Crohn's disease, Stricture, Surgery, Case report

## Abstract

**Introduction and importance:**

Stricture formation is a well-known Crohn's disease consequence that usually results from recurrent cycles of inflammation and healing and primarily affects the small intestine.

**Case presentation:**

In this report, we describe the case of a 35-year-old male with an 18-year history of Crohn's disease complicated by long-kinked ileal stricture who presented with a 3-month history of subacute small intestinal obstruction diagnosed with MR enterography and underwent failed medical treatment.

**Clinical discussion:**

The patient, a male showing signs of wasting due to a prolonged subacute small intestinal obstruction, underwent an MR enterography which revealed a 6-cm kinked ileal stricture. Intraoperative observations included a significantly dilated small intestine proximal to the stricture and a collapsed distal small bowel. Following resection, the patient experienced a smooth recovery with marked improvement.

**Conclusion:**

When there are clear indications for the surgical resection of a stenosed bowel segment caused by Crohn's disease, it is advisable to proceed with the surgery promptly, with a preference for side-to-side stapled anastomosis.

## Highlights

• A 35-year-old male has been experiencing subacute small intestinal obstruction, following an 18-year history of Crohn's disease.
• MRI enterography is the preferred diagnostic method for defining the characteristics of a stricture.
• Failure of medical treatment should prompt endoscopic or surgical intervention.
• When medical treatment fails, it should prompt consideration of endoscopic or surgical intervention.

## 1 Introduction

Stricture formation is a well-known complication of Crohn's disease (CD), typically arising from recurrent cycles of inflammation and healing, and it predominantly affects the small intestine. About 20 % of patients are affected at diagnosis, and it may concern up to 50 % of patients throughout their lives [1]. Strictures are the leading cause of hospital admissions and surgical interventions among individuals with Crohn's disease [2]. Symptoms such as nausea, vomiting, postprandial abdominal pain, distention, and dietary restrictions may suggest a stricture, but they do not strongly correlate with the actual presence of strictures observed in imaging or endoscopy. Furthermore, the severity of small bowel strictures does not consistently match the presence of obstructive symptoms. This suggests that symptoms alone are not reliable for diagnosing strictures, thus additional testing is required for an accurate diagnosis [3]. When a stricture contains an inflammatory component, anti-inflammatory therapy can be advantageous. Conversely, in cases of a purely fibrotic stricture, such treatment is usually ineffective. Instead, endoscopic balloon dilation (EBD) or surgical intervention are the preferred management approaches [4]. This report presents a case of Crohn's disease that was complicated by severe stenoses, necessitating surgical resection as the best option.

## 2 Case presentation

A 35-year-old male has been experiencing subacute small intestinal obstruction for the past three months. Diagnosed with Crohn's disease at the age of 17 following nonspecific abdominal complaints, significant weight loss, and the emergence of complex perianal fistulae, he underwent multiple endoscopies and biopsies to confirm the diagnosis. The patient commenced treatment with 5-aminosalicylic acid and azathioprine. The patient achieved full remission while on maintenance therapy for approximately 10 years and successfully gained weight, reaching 88 kg.

**Fig. 1:**
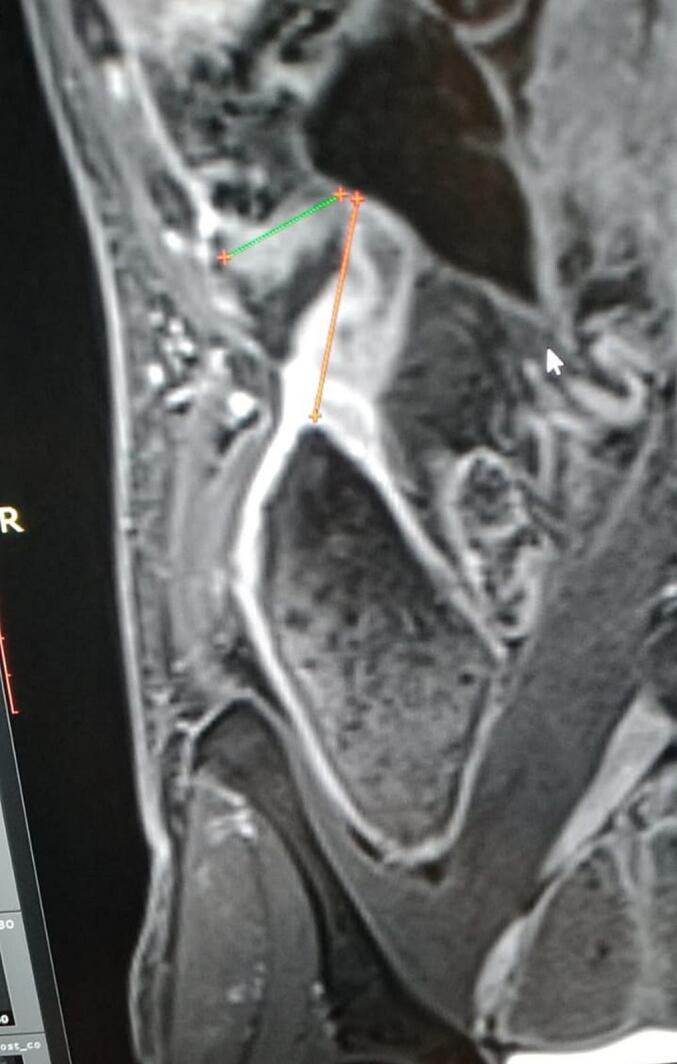
The MR enterography coronal section revealed severe ileal stenoses with kinking (indicated by arrows) and significant dilatation of the small intestine.

The patient is not a smoker, not an alcohol consumer, and had social embarrassment due to his gastrointestinal symptoms such as flatulence and borborygmi. Six years prior, he experienced acute small bowel obstruction at multiple sites, resulting in a 20-day hospital admission. The temporary addition of hydrocortisone to his treatment led to significant but incomplete improvement. He frequently suffered from abdominal pain, indigestion, and weight loss. Three months earlier, he experienced frequent vomiting, borborygmi, and abdominal distension. His weight dropped to 53 kg. MRI enterography revealed a 6-cm stricture and kinking at the terminal ileum, likely longstanding, associated with severe proximal medial small bowel dilatation, abdominal distension and collapse of the colon (Fig. 1).

**Fig. 2:**
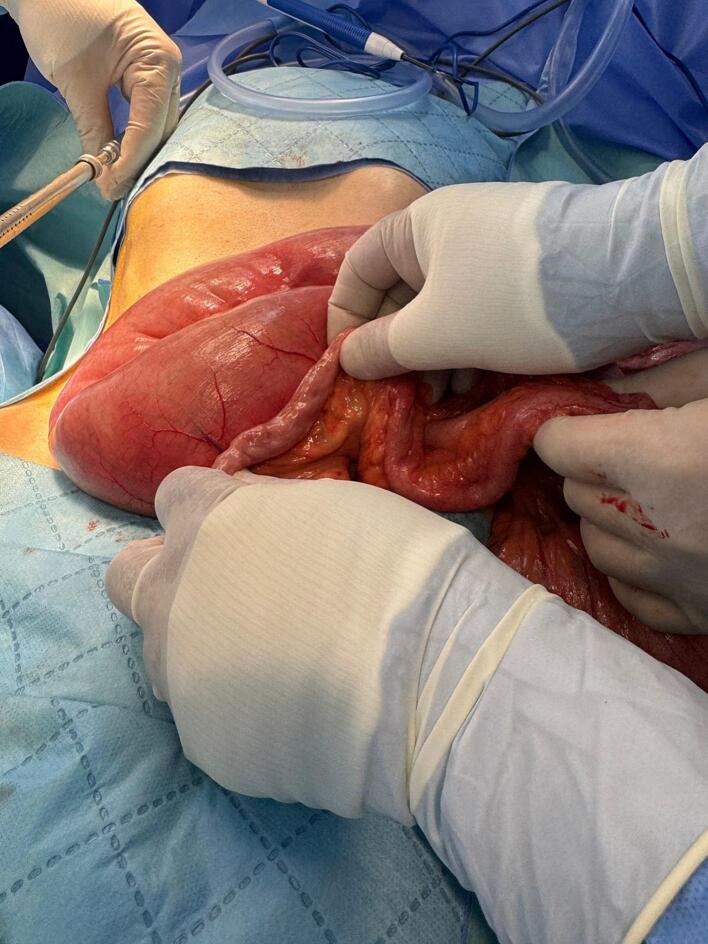
Massive dilatation of the proximal small intestine and collapsed distal small intestine.

**Fig. 3:**
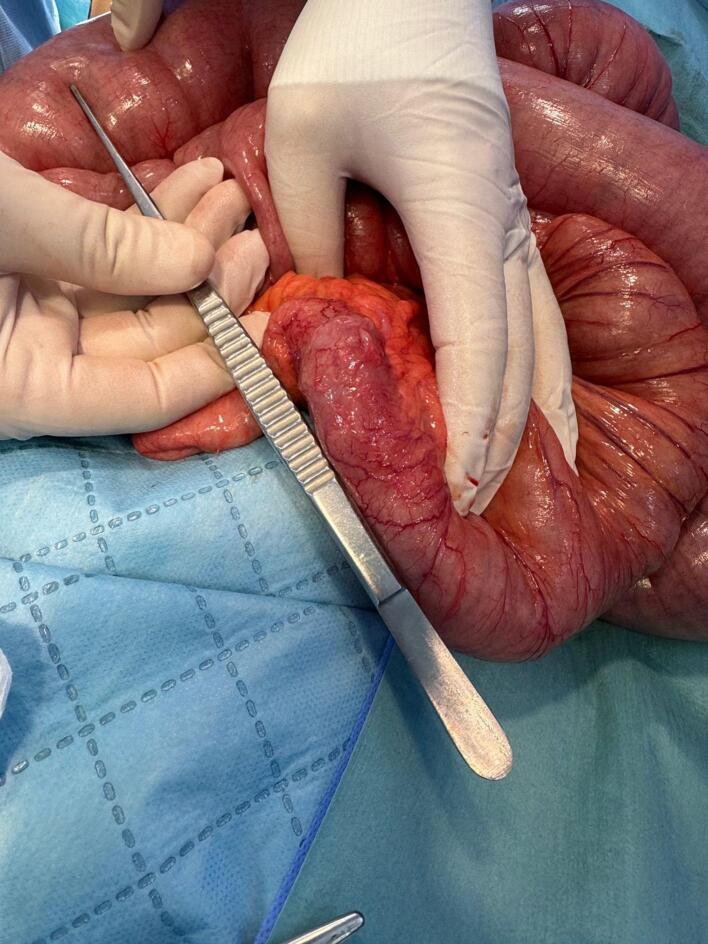
The area of elongated, kinked ileal stenoses indicates the transition from the dilated to the collapsed segments of the small intestine.

The patient was prescribed a 40 mg intravenous adalimumab injection to be administered bi-weekly. Despite this, there was no improvement, leading to the decision to proceed with surgery. The patient underwent a laparoscopic resection attempt, which was converted to an open resection due to inadequate workspace. During the operation, extensive dilation of the small intestine was observed, located 40 cm proximal to the ileocecal valve, along with the collapse of the distal segment (Fig. 2), attributed to a 6 cm area of stenosis in the ileum (Fig. 3). There were no fluid collections or adhesions to the abdominal wall or internal fistulas. Stapled resection of the stenosed ileal segment, followed by a side-to-side anastomosis. Over time, the patient showed gradual improvement; his symptoms resolved, and he achieved a weight of 68 kg after three months of follow-up.

This case report article was prepared according to the regulations of the Ethical Committee and is approved by the Institutional Review Board (IRB) number (1662024). The patient has provided informed consent for publication of the case. The research has been reported in line with the SCARE criteria [5].

## 3 Discussion and conclusion

Crohn's disease is a chronic inflammatory disorder of the entire gastrointestinal tract, characterized by transmural inflammation and a varied, asymmetric, segmental pattern. The global incidence and prevalence of Crohn's disease are on the rise, with annual increases in incidence rates ranging between 4 % and 15 % over the last thirty-five years [6]. In small bowel disease, the affected segment typically exhibits thickening along the mesenteric edge of the bowel and fat wrapping around the sides of the intestinal wall [7]. The pathogenesis of Crohn's disease strictures, while not completely understood, involves a complex interaction of inflammatory and non-inflammatory pathways that contribute to the development of fibro stenosis, or stricture [4].

MR enterography, utilized for our patient, remains the preferred diagnostic technique as it provides a detailed assessment of the stricture and its elements using multiple sequences, with and without contrast enhancement, and avoids the use of ionizing radiation [8]. In the context of the literature review, it is important to note that the CONSTRICT group has developed a consensus-based definition of stenosis for imaging purposes in clinical settings, which extends beyond clinical trials. For a diagnosis, at least two of the following characteristics must be present: localized luminal constriction (>50 % narrowing), thickening of the intestinal wall, and pre-stenotic dilation (usually >3 cm). However, for clinical research purposes, all three criteria must be met [3]. For our patient, all the required criteria have been met.

Hospitalization is typically necessary for acute small-bowel obstruction. Although corticosteroid therapy is frequently used in such cases, the evidence supporting its effectiveness is still limited [1]. While methotrexate is utilized in treating Crohn's disease, its efficacy has not been assessed in cases of Crohn's disease with stricture [9]. Adalimumab underwent evaluation in a single-arm, multicenter, prospective observational cohort study involving 97 patients with small bowel stricturing Crohn's disease (the CREOLE study). At the 24-week mark, 64 % of patients remained on therapy without the need for steroids, endoscopic balloon dilation, or surgery. After four years, 29 % of patients continued successful treatment with adalimumab, while approximately half required surgery within this period [10]. Vedolizumab and Ustekinumab have been proven safe and effective for treating Crohn's disease; however, data regarding their impact on strictures is not available [11].

Anti-inflammatory therapy, endoscopic procedures, surgery, or a combination thereof are frequently required. In a recent retrospective cohort of patients with CD who presented with acute small intestinal obstruction, 22.5 % required surgery within 6 months. Female gender, BMI <25, penetrating disease, impacted segment length, and CT scan intestinal wall enhancement were all related with surgery within 6 months [12]. The probability of patients with Crohn's disease requiring surgical resection within 15 years of diagnosis stands at 70 % [13], as exemplified by our patient. Surgical intervention is recommended for symptomatic structuring diseases that do not respond to medical or endoscopic treatments, as well as for conditions linked with potential or established malignancy, or penetrating diseases that involve complex fistulas [14]. Endoscopic balloon dilation (EBD) is suitable for endoscopically accessible, non-angulated, and short strictures under 5 cm in length. It should not be performed if contraindications, such as penetrating disease, abscesses, or malignancy, are present [15].

The decision to proceed with surgical intervention is influenced by the type of disease, the pattern of stricture, patient preferences, the presence of complications like abscesses, phlegmon, or internal penetrating disease, and the consensus of an interdisciplinary team [16]. In the case of our patient, the preference of both the multidisciplinary team and the patient was to opt for surgical resection. This decision was due to the persistent nature of the obstructive disease, which was accompanied by malnutrition, considerable weight loss, a diminished quality of life, and a long, kinked stricture that was unresponsive to medical therapy.

The two most common surgical treatments for stricture disease are segmental excision and stricturoplasty [17]. Segmental resection entails removing the affected segment, usually followed by the creation of an end-to-end, end-to-side, or side-to-side anastomosis [14].

A meta-analysis comparing 396 stapled side-to-side anastomoses with 425 hand-sewn end-to-end anastomoses found that the stapled method was superior across all endpoints. This included overall postoperative complications (OR 0.54, 95 % CI 0.32–0.93), anastomotic leak (OR 0.45, 95 % CI 0.20–1.00), recurrence (OR 0.20, 95 % CI 0.07–0.55), and reoperation due to recurrence (OR 0.18, 95 % CI 0.07–0.45) [18]. A network meta-analysis encompassing 11 trials and 1113 patients has confirmed that stapled side-to-side anastomosis is superior regarding overall complications, clinical recurrence, and reoperation rates for recurrence [19].

Finally, surgical resection, when performed at the time of diagnosis or early in the disease course, can result in extended periods of clinical remission, reduced rates of long-term surgeries, and a lesser reliance on steroids and biologic therapies during subsequent follow-up [20].

## Author contribution

M.N. Designed the study, and analyzed data analysis, and paper writing.

A.Y Manuscript writing and participated in the design of the study.

S.S. Prepared the images and corresponding data of the case.

B.A. Critically evaluated the manuscript and participated in writing the manuscript.

All authors read and approved of the final manuscript.

## Informed consent

Written informed consent was obtained from the patient for publication of this case report and accompanying images. Surgical choices, benefits, and side effects are explained to the patient. A copy of the written consent is available for review by the Editor-in-Chief of this journal on request.

## Ethical approval

This research is approved by the Institutional Review Board (IRB) in the School of Medicine at Mutah University. Number 1662024

## Guarantor

Dr. Mohammad Nebih Nofal (first author) is the guarantor for this work.

## Research registration number

Not applicable.

## Funding

This research is not funded from any source.

## Conflict of interest statement

The authors disclose no conflict of interest.

## Data Availability

All data analyzed or generated during this case report are included in this article. Further enquiries can be directed at the corresponding author.
